# Improved Photodynamic Inactivation of Resistant *Nakaseomyces glabrata* Yeasts and Biofilms Mediated by ZnTE-2-PyP^4+^ Porphyrin Combined with Silver Nanoparticles

**DOI:** 10.1021/acsomega.5c10859

**Published:** 2026-02-18

**Authors:** Geyse S. de Lima, Sueden O. Souza, Jacqueline C. Bueno-Janice, Bruno L. Raposo, Franz A. G. dos Santos, Rejane P. Neves, Beate S. Santos, Jose F. Sarmento-Neto, Julio S. Reboucas, Paulo E. Cabral Filho, Adriana Fontes

**Affiliations:** † Departamento de Biofísica e Radiobiologia, Universidade Federal de Pernambuco, Recife 50670-901, PE, Brazil; ‡ Centro de Lasers e Aplicações, Instituto de Pesquisas Energéticas e Nucleares (IPEN-CNEN/SP), São Paulo 05508-000, SP, Brazil; § Departamento de Micologia, 28116 Universidade Federal de Pernambuco, Recife 50760-420, PE, Brazil; ∥ Departamento de Ciências Farmacêuticas, Universidade Federal de Pernambuco, Recife 50740-520, PE, Brazil; ⊥ Departamento de Química, 28097 Universidade Federal da Paraíba, João Pessoa 58051-900, PB, Brazil

## Abstract

*Nakaseomyces glabrata* is a high-priority fungal pathogen due to its incidence and antifungal resistance. Photodynamic inactivation (PDI) can offer a promising approach against resistant *N. glabrata*, particularly when the advantageous photophysical properties of Zn­(II) porphyrins can be enhanced by the plasmonic effect of metal nanoparticles (NPs). Herein, the association of ZnTE-2-PyP^4+^ porphyrin (ZnP-ethyl) with AgNPs (stabilized with polyvinylpyrrolidone, PVP) in PDI against yeasts and biofilms of resistant *N. glabrata* strains was investigated. AgNPs/ZnP-ethyl (NE) systems were prepared, and physicochemical characterizations indicated the interaction and spectral overlap between AgNPs and ZnP-ethyl, prerequisites for harnessing the plasmonic effect. Moreover, AgNPs had a minimal effect on ZnP-ethyl fluorescence lifetime. To investigate the role of AgNPs and strain susceptibility in PDI, yeast interactions with NE and ZnP-ethyl were assessed by fluorescence microscopy, which indicated that, in general, AgNPs facilitated the internalization of ZnP-ethyl by cells. Interestingly, HGV14 yeasts, which were unable to form biofilm, exhibited the lowest susceptibility to NE-PDI, likely due to reduced cell interaction relative to the other strains. PDI using ZnP-ethyl alone at 1.5 μM reduced HGV11 and HGV20 yeasts by 1 log_10_, whereas NE-mediated PDI eradicated cells using 4–5-fold lower ZnP-ethyl concentrations. In biofilms, NE-PDI reduced the viability by ∼75% and induced high cell death to a much greater extent compared to other irradiated groups. Therefore, NE boosted the PDI of yeasts and biofilms across all resistant *N. glabrata* strains, likely driven by plasmonic effect and enhanced cell interaction promoted by PVP-AgNPs, making NE-PDI a promising strategy for combating resistant microorganisms.

## Graphical abstract

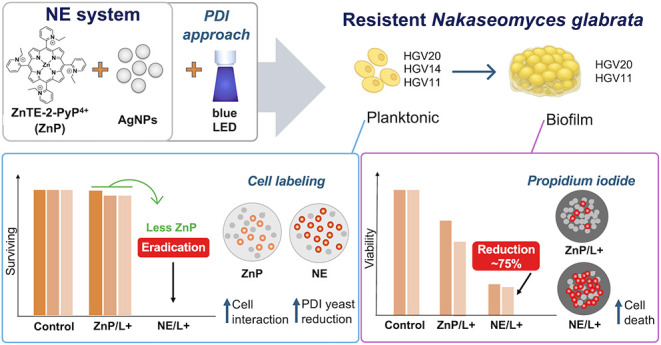

## 1 Introduction

Fungal infections have become a major global health threat, driven by their association with increased morbidity and mortality rates.^1,2^ Over the past 30 years, progress has been made in the development of antifungal drugs. However, the growing resistance to existing treatments underscores the urgent need for new therapeutic options. Indeed, antimicrobial resistance remains one of the most critical challenges in modern medicine, as the rise of resistant microorganisms reintroduces the threat of infections that are unresponsive to current therapeutic approaches.^3,4^

In light of the concerning emergence of resistant fungal strains, the World Health Organization (WHO) released a list of priority fungal pathogens to guide research, surveillance, and resource allocation in the fight against mycoses.^5^ *N. glabrata* is classified within the high-priority group on this list. This species (formerly *Candida glabrata*) has gained increasing clinical relevance as the second most common cause of fungal infections (after *Candida albicans*).^6,7^ Additionally, *N. glabrata* strains can present reduced susceptibility to available antifungal drugs,^8^ coupled with a high ability to suppress the immune response and adapt to changes in environmental conditions.^9^ This species can also develop important resistance mechanisms, such as biofilm formation, which shields fungal cells and creates additional barriers to effective treatment.^6,10^

It is worth noting that excessive exposure of the population to available antifungal drugs contributes to the selection of resistant isolates, which can proliferate and cause infections that are more difficult to eradicate.^3^ Given this, developing therapies to expand the antifungal arsenal and reduce the risk of emerging resistance is paramount to managing mycoses worldwide.

In this regard, photodynamic inactivation (PDI) has emerged as an attractive and alternative approach to managing superficial microbial infections. In PDI, a photosensitizer (PS) is excited by light at a wavelength that overlaps with its absorbance, in the presence of molecular oxygen, leading to the generation of reactive oxygen species (ROS) that cause microbial cell death.^11,12^ PDI can be controlled both spatially and temporally, reducing the risk of systemic off-target effects, commonly associated with antifungal drugs.^13^ Moreover, studies suggest that as PDI simultaneously acts on multiple subcellular components, the development of anti-PDI resistance is unlikely.^14^ Additionally, medical devices can harbor fungal colonization, and PDI can also be used to decontaminate these surfaces, helping to prevent infections.^15,16^

Our research group has been actively contributing to the field of PDI, investigating hydrophilic cationic Zn­(II) porphyrins (ZnPs) as PSs.^17–21^ ZnPs exhibit promising features for PDI, owing to their low dark toxicity and efficiency in ROS generation.^11^ The chemical structure of ZnPs can also be tailored to modulate their lipophilicity and ionic character, tuning their interaction with cellular structures.^11^ Additionally, photodynamic treatment with ZnPs has shown minimal effects on mammalian cells.^17,19^

Metal nanoparticles (NPs) can be engineered and combined with PSs to enhance PDI.^22^ When excited by light at a wavelength resonant with their extinction band, metal NPs undergo localized surface plasmon resonance (LSPR), in which conduction-band electrons collectively oscillate at the metal-surrounding-medium interface in response to an external electromagnetic field, giving rise to the plasmonic effect. This phenomenon can, for example, amplify the local electromagnetic field around the nanostructure,^23^ which can boost the PS interaction with light, thereby increasing ROS production and PDI performance.^24,25^ In addition, plasmon-to-PS energy transfer, which can also assist the PS excitation, and NP-facilitated electron transfer to the PS, which can support the generation of reactive intermediates, may also contribute to ROS generation and microbial photokilling.^25^ The mechanisms underlying PDI enhancement via the plasmonic effect remain under elucidation.

Therefore, motivated by the rise of resistant fungal strains and the challenges posed by biofilms to current antifungal treatments and aiming to delve deeper into the metal-assisted PDI approach, this study investigated the application of Zn­(II) *meso*-tetrakis­(*N*-ethylpyridinium-2-yl)­porphyrin (ZnTE-2-PyP^4+^, ZnP-ethyl) combined with AgNPs (stabilized with polyvinylpyrrolidone, PVP) in PDI of yeasts and biofilms of *N. glabrata* resistant strains. To the best of our knowledge, few studies have been conducted on the use of AgNPs in antifungal PDI so far, particularly against resistant strains and biofilms, with research focusing primarily on *C. albicans*.^22,26^ We hope our findings help expand the arsenal for tackling resistant strain-associated mycoses and contribute to advancing knowledge on the use of metal NPs in PDI strategies.

## 2 Experimental Procedures

### 2.1 *N. glabrata* Strains and Culture Conditions

Clinical isolates of *N. glabrata* (HGV11, HGV14, and HGV20), obtained from URM Culture Collection (Department of Mycology, Federal University of Pernambuco), were used in this study. HGV11 is resistant to itraconazole and voriconazole, HGV14 is resistant to caspofungin, and HGV20 is resistant to anidulafungin and caspofungin (antibiogram profile in the Supporting Information, Table S1). Working cultures were streaked from −80 °C stocks onto Sabouraud Dextrose Agar (SDA, HiMedia) and incubated at 37 °C for 24 h and then maintained at 4 °C. For each experiment, one loop-full of culture was inoculated in 4 mL of Sabouraud Dextrose Broth (SDB, Neogen) and incubated at 37 °C overnight. The yeasts were then centrifuged and washed once with phosphate-buffered saline (PBS, 1×, pH 7.4) at 700*×g* for 90 s (MiniSpin—Eppendorf), and resuspended to a final concentration of approximately 1 × 10^7^ colony-forming units per milliliter (CFU/mL). The cell concentration was assessed at the optical density at 540 nm (OD_540_) in a spectrophotometer (SPECTROstar Nano, BMG Labtech). The cell density was standardized using a Neubauer chamber.

### 2.2 Biofilm Formation

Overnight liquid cultures were grown and washed as described in Section 2.1. Washed cells were resuspended in PBS and diluted to approximately 1 × 10^7^ CFU/mL in RPMI 1640 medium (Sigma-Aldrich, phenol red-free) supplemented with 20 mM HEPES (Sigma-Aldrich). The cell suspension (100 μL) was transferred to flat-bottom 96-well microplates (KASVI–K12–096) and incubated for 90 min at 37 °C, under 75 rpm shaking (Solab, SL223). The microplate wells were gently washed twice with 200 μL of PBS and incubated with 200 μL of the same supplemented RPMI medium for 48 h to obtain mature biofilms.^19^ An initial screening of the yeast strains using the 3-(4,5-dimethylthiazol-2-yl)-2,5-diphenyltetrazolium bromide (MTT) assay revealed a limited ability of HGV14 to form biofilms (OD_570_ similar to the blank), and thus, this strain was not included in PDI assays of biofilms.

### 2.3 Synthesis and Characterization of ZnP-Ethyl and AgNPs

ZnP-ethyl was obtained by alkylation of the precursor Zn­(II) *meso*-tetrakis­(2-pyridyl)­porphyrin^27^ presenting spectral and chromatographic characteristics identical to those previously reported.^11,28^ The concentration of the ZnP-ethyl aqueous stock solution was determined spectrophotometrically, in water, using the reported molar extinction coefficient for the Soret band (ε_425.5 nm_ = 288,403 M^–1^ cm^–1^).^28^

AgNPs were synthesized in aqueous medium,^29^ via the chemical reduction of silver nitrate (AgNO_3_, 25.0 mL, 0.1 mM, Sigma-Aldrich) with trisodium citrate (Na_3_C_6_H_5_O_7_, 1.5 mL, 30 mM, Dinâmica), under vigorous stirring for 5 min at room temperature (RT, approximately 25 °C). Subsequently, a solution of PVP (1.5 mL, 0.7 mM, Sigma-Aldrich, MW 29,000) was added, followed by the incorporation of sodium borohydride (NaBH_4_, 0.15 mL, 100 mM, Sigma-Aldrich), which had been refrigerated at 4 °C, resulting in a change of the suspension color from clear to translucent yellow. The suspension was stirred continuously for 30 min. AgNPs were characterized using ultraviolet–visible (UV–vis) spectroscopy (SPECTROstar Nano, BMG Labtech), Zeta (ζ) potential analysis (Zetasizer NanoZS, Malvern), and transmission electron microscopy (TEM, FEI Tecnai Spirit Bio-Twin, 120 kV).

### 2.4 Preparation and Characterization of NE Systems

To obtain the AgNPs/ZnP-ethyl (NE) systems, the suspension of AgNPs was filtered by centrifugation (2495×*g* for 10 min, Universal Centrifuge 320R–Hettich Zentrifugen) using 10 kDa spin columns (Vivaspin, Cytiva), then resuspended to half of the initial volume. This procedure was also performed to remove excess residual reagents from the synthesis. Working solutions of ZnP-ethyl were freshly prepared in ultrapure water from a concentrated stock solution. After, they were mixed with the filtered AgNPs suspension (approximately 10^12^ NPs/mL)^20^ to achieve the desired final Zn­(II) porphyrin concentration at 1:4 or 4:1 volume ratios (NPs/PS). The final volume was kept constant regardless of the Zn­(II) porphyrin concentration and NPs/PS ratios used. NE systems were prepared with twice the Zn­(II) porphyrin concentration applied in the PDI assays. After preparation, the systems were gently mixed (Gilson–Rota-mini plus) for 48 h in the dark before use.^20^

NE systems were examined by UV–vis (UV-1800, Shimadzu) and fluorescence spectroscopies (LS55, PerkinElmer). Zeta (ζ) potential analysis of NE was also performed (PS at 3 μM, about twice the highest concentration tested in PDI, Zetasizer NanoZS, Malvern). Fluorescence lifetime (τ) measurements for ZnP-ethyl and NE were obtained by using the time-correlated single photon counting (TCSPC) mode of a FluoroLog QM spectrofluorometer (Horiba Jobin Yvon). Samples (at 1.2 μM of porphyrin) were excited at 390 nm with a pulsed diode laser (DeltaDiode) in a quartz cuvette at RT. The instrument response function (IRF) for scattering was calibrated using a nonfluorescent aqueous suspension of colloidal silica beads (Ludox 40%, Sigma-Aldrich) to account for the system’s temporal dispersion and enable accurate measurement of τ. The measurements were analyzed using FelixFL analysis software to obtain τ values, based on the Durbin–Watson and χ^2^ statistical parameters.

### 2.5 Cell Interaction Analyses

The interaction between the ZnP-ethyl or NE system with *N. glabrata* yeast cells was studied using confocal microscopy, exploring the intrinsic fluorescence of the Zn­(II) porphyrin. Cells (approximately 1 × 10^7^ CFU/mL) were incubated for 60 min at 37 °C on microscopy imaging chambers (CELLview, Greiner Bio-One). After incubation, the chambers were washed twice to remove nonadhered cells and then incubated with the ZnP-ethyl or the NE system (1:4 v/v) at a final porphyrin concentration of 5 μM for 10 min (same incubation time used in PDI assays with cells in suspension). This concentration was selected to ensure proper visualization of the Zn­(II) porphyrin fluorescence. After incubation, the chambers were washed twice again to remove excess ZnP-ethyl or NE system and then analyzed through multispectral confocal fluorescence microscopy (FV1000, Olympus) with excitation at 473 nm, using a 63× oil immersion objective lens (numerical aperture 1.35), and collecting the emission at 670/60 nm. The same acquisition parameters were maintained across the analyses. Heatmaps of fluorescence intensity were generated in FIJI (v. 2.16.0),^30^ which was also employed to measure cell sizes.

### 2.6 Photodynamic Treatment of *N. glabrata* Planktonic Cells

PDI assays with planktonic cells (yeasts in suspension) were conducted in 96-well microplates. The samples were illuminated from above with a blue LED (410/20 nm, LEDbox, Biolambda), as the absorption band of ZnP-ethyl and the plasmon band of AgNPs fall within this wavelength range. Irradiated (L+) and dark (L−) groups were the following: Control (without any treatment): NE/L–, ZnP-ethyl/L–, NE/L+, ZnP-ethyl/L+, AgNPs/L+, and L+ only. The ZnP-ethyl concentration in NE systems ranged from 0.3 to 2 μM, depending on the strain under the analyses. The NP concentration in the AgNPs/L+ group was equivalent to that in the NE 4:1 system (ultrapure water was used in place of porphyrin). The assays were conducted with a 1:1 v/v ratio, using 100 μL of *N. glabrata* cell suspension (at approximately 1 × 10^7^ CFU/mL) and 100 μL of the compounds (NE, ZnP-ethyl only, or AgNPs only) or PBS (Control). L+ groups were incubated with the compounds for 10 min at RT and then irradiated for 3 min (corresponding light doses in the Results and Discussion Section).^19^ Control and L– groups were incubated for 13 min. Irradiated and nonirradiated samples were serially diluted in PBS, and 10 μL of each dilution was added to a Petri dish containing SDA, then incubated at 37 °C for 24 h for CFU counting, using the methodology proposed by Jett et al.^31^ At least three independent experiments were conducted, each with three replicates per group.

### 2.7 Photodynamic Treatment of *N. glabrata* Biofilms

PDI assays were also performed on *N. glabrata* HGV20 mature biofilms. The groups were: Control (without any treatment): NE/L–, ZnP-ethyl/L–, AgNPs/L–, NE/L+, ZnP-ethyl/L+, AgNPs/L+, and L+ only. Effects of L+ groups on HGV11 mature biofilms were also investigated. The biofilms were gently washed with 200 μL of PBS, incubated with the compounds (NE, ZnP-ethyl only, AgNPs only, or PBS) for 20 min, and then irradiated using the same parameters applied to the yeasts. Control and L– groups were incubated for 23 min. After photodynamic treatments, the compounds were gently removed, and the biofilms were incubated with 200 μL of MTT (0.5 mg/mL, in the same supplemented RPMI medium used for biofilm formation, Sigma-Aldrich) for 2 h at 37 °C. The liquid was removed, and 200 μL of dimethyl sulfoxide (DMSO, ACS Cientfica) was added to the biofilms, which were incubated for 15 min at RT and 75 rpm (Solab SL223). After incubation, 150 μL from each group was transferred to a new 96-well microplate for OD_570_ measurement (SPECTROstar, BMG Labtech). The MTT assay was performed to evaluate the effect on viability, which was inferred from mitochondrial-dependent metabolic activity, as only viable cells can reduce MTT to formazan. The MTT assay was performed in quadruplicate for each group across at least four independent experiments.

To complement the MTT assay, the photodynamic effects in biofilms were also analyzed by propidium iodide (PI) staining using confocal fluorescence microscopy. For this, biofilms were grown in microscopy imaging chambers (CELLview, Greiner Bio-One) and PDI mediated by NE, ZnP-ethyl alone, and AgNPs alone was performed, in accordance with the previously described procedures. Subsequently, biofilms were washed with PBS to remove any excess compounds, and PI (V13245, Thermo Fisher Scientific) was added at 1 μg/mL to the chambers, which were kept in the dark at RT for 15 min.^19^ Following the incubation period, biofilms were washed to eliminate any residual PI and then examined through multispectral confocal fluorescence microscopy (FV1000, Olympus) with excitation at 473 nm and using a 63× oil immersion objective lens (numerical aperture 1.35). The same acquisition parameters were maintained across analyses and were chosen so that porphyrin emission was not detected; for this, samples incubated with the PS in the dark were used as controls, and PI emission was collected at 710/60 nm. At least three representative images were acquired for each sample, and the assay was performed in two independent experiments.

### 2.8 Statistical Analysis

The data were analyzed using GraphPad Prism 8 software to identify statistical differences. The Shapiro–Wilk test was performed to assess the normality of the data distribution. The Mann–Whitney test was then used to compare differences between the experimental groups, with statistical significance set at *p* < 0.05.

## 3 Results and Discussion

### 3.1 Characterization of AgNPs and NE Systems

AgNPs were synthesized by chemical reduction, exhibiting a predominantly near-spherical shape with an average size of 14.2 nm (Figures 1A and S1 from the Supporting Information). Moreover, NPs displayed a plasmon peak at approximately 410 nm, which overlaps with the Soret band of the Zn­(II) porphyrin (Figure 1B) as well as with the wavelength range of the light source used in PDI assays. This spectral overlap is a prerequisite for enhancing PDI via the plasmonic effect. These findings are in accordance with the literature.^29^

**Figure 1:**
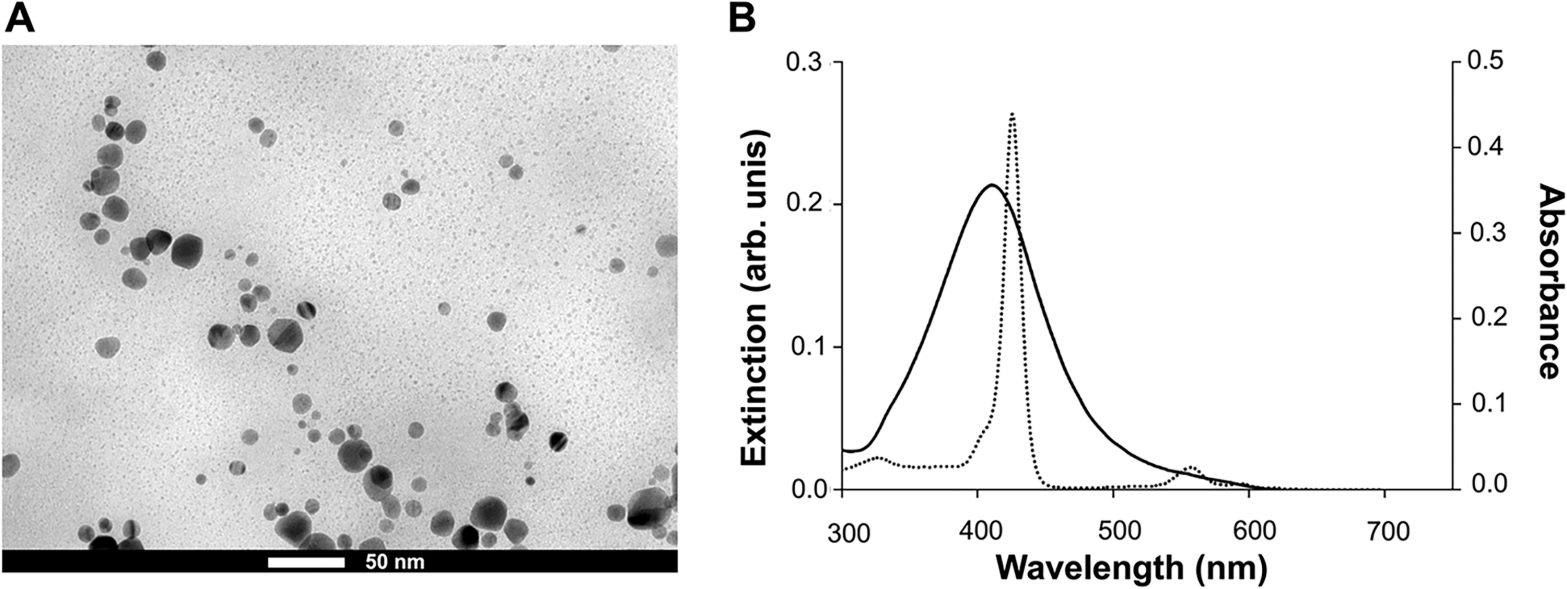
(A) Representative TEM image of AgNPs, scale bar = 50 nm. (B) Extinction spectrum of AgNPs (solid line) showing a plasmon peak at approximately 410 nm, and absorption spectrum of ZnP-ethyl acquired in water (dotted line).

Additionally, AgNPs exhibited a negative ζ potential value of −43 ± 3 mV due to the anionic PVP polymer, which shifted to −22 ± 3 and −24 ± 2 mV in the NE systems (for 1:4 and 4:1 v/v ratios, respectively) following Zn­(II) porphyrin addition. This decrease in the NP negative charge indicates an interaction between AgNPs and ZnP-ethyl, following a similar pattern to that observed for other NPs and PSs,^32,33^ and corroborating our previous results.^20^ The interaction suggests that ZnP-ethyl and PVP-AgNPs are in proximity, which is also relevant for exploring the LSPR to intensify photodynamic outcomes.

Regarding the optical behavior, there were just subtle changes in UV–vis absorption and emission profiles of NE compared to data of the Zn­(II) porphyrin alone (data not shown), as also previously reported by us.^20^ Fluorescence decay measurements (Supporting Information, Figure S2) were best fitted by a biexponential model; however, the contribution of the longer-lived component (τ_2_) was minimal (about 0.2%). Thus, our analysis focused primarily on the dominant lifetime component, τ_1_. ZnP-ethyl exhibited a τ_1_ of 1.72 ns, which, to the best of our knowledge, is reported here for the first time. For the NE systems (4:1 and 1:4), similar τ_1_ values were observed for both ratios (1.68 ns). The small decrease in τ for NE suggests that the AgNPs had minimal influence in fluorescence decay rate of ZnP-ethyl under the conditions applied. On the other hand, compounds that significantly shorten fluorescence τ of PSs may not favor ROS production, as intersystem crossing is also diminished under such conditions.^34^ In our previous work, we reported enhanced ROS generation for NE compared to ZnP-ethyl alone.^20^ Thus, we believe that the favored intersystem crossing and the enhanced PS excitation rate due to the locally amplified electromagnetic field near NPs are likely the main contributors to the higher ROS levels observed for NE. Nevertheless, some involvement of plasmon-to-PS energy transfer and/or NP-facilitated electron transfer cannot be excluded.^25,34,35^ The role of metal NPs in this context remains complex and is not yet fully understood, encouraging continued investigation.

### 3.2 Cell Interaction Analyses

The interaction between ZnP-ethyl or NE with *N. glabrata* yeast cells was studied by using confocal fluorescence microscopy (Figure 2). Panels A1 and A3 show images of *N. glabrata* HGV20 yeast cells, presenting the overlay of bright-field and fluorescence channels after incubation with ZnP-ethyl only (A1) or NE (A3). The more intense red color in panel A3 relative to panel A1 indicates that a more effective cell labeling was visualized by applying the NE system. Panels A2 and A4 display the fluorescence heatmaps, providing an alternative view of the differences in labeling promoted by ZnP-ethyl alone (A2) or NE (A4). A heatmap is a visual representation that uses a color gradient to depict fluorescence signal intensity, here ranging from low (dark blue) to intermediate (green/yellow) and high (white). Panel A4 shows cells in light green and yellow colors, indicating higher labeling, compared to panel A2, which shows cells in dark and light blue.

**Figure 2:**
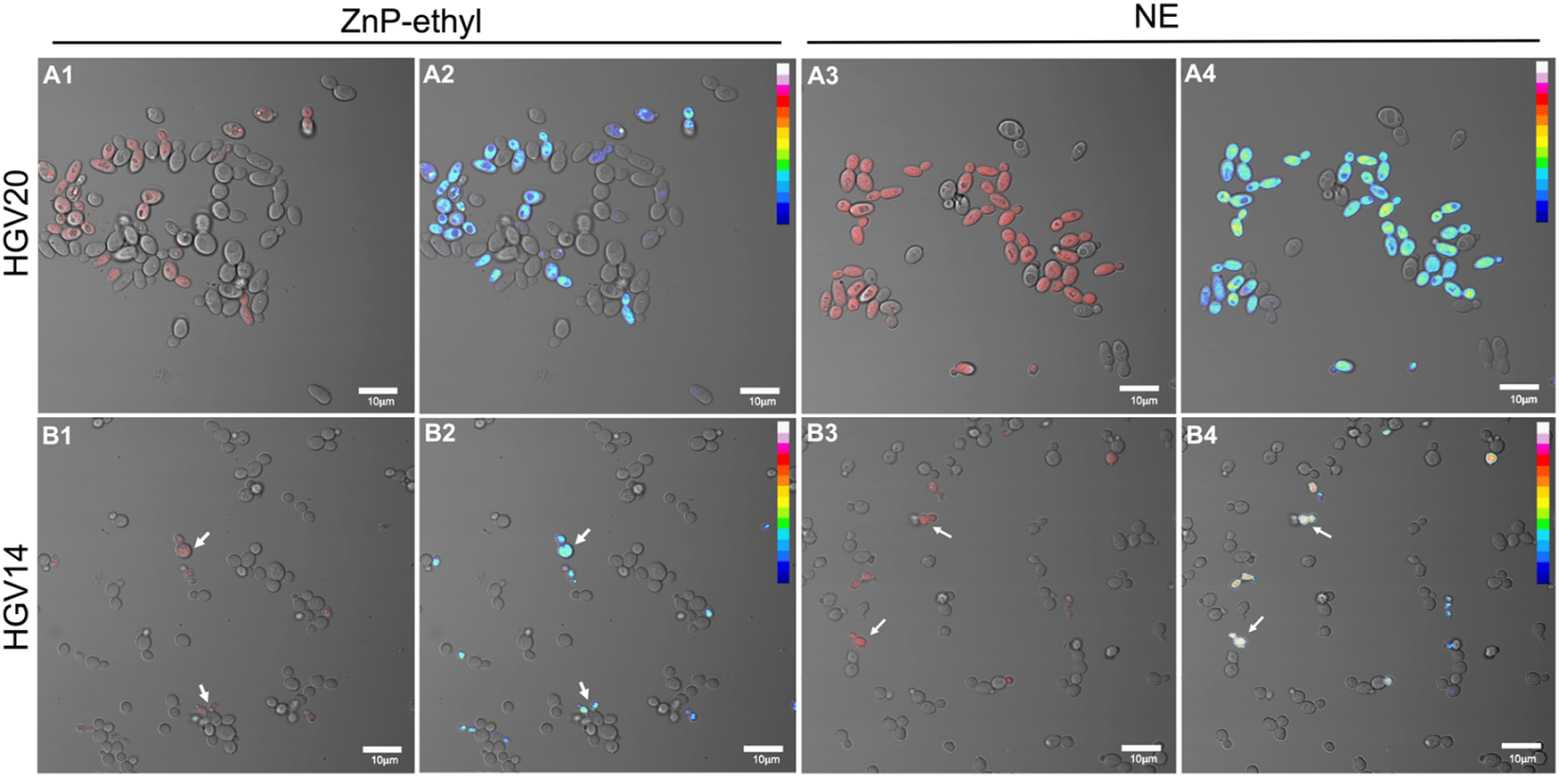
Representative confocal microscopy images of HGV20 (A1–A4) and HGV14 (B1–B4) *N. glabrata* yeast cells, showing the overlay of bright-field and fluorescence channels after 10 min of incubation with ZnP-ethyl (A1, B1) or NE 1:4 system (A3, B3). In A2, A4, B2, and B4 are the corresponding fluorescence heatmaps, where the signal intensity increases from dark blue to white. The arrows indicate some labeled HGV14 cells. Scale bar = 10 μm.

Based on our results, we suggest that delivery mediated by AgNPs drove an increased cellular uptake of ZnP-ethyl. Indeed, surface-modified AgNPs have been explored as delivery systems, for example, in gene therapy.^36,37^ A similar behavior was observed in our previous study with *C. albicans* (ATCC 90028), where the cell interaction with porphyrin was also facilitated by the NPs. Negligible labeling was, however, detected in *C. albicans* strain when it was incubated with ZnP-ethyl alone,^20^ indicating that ZnP-ethyl was better internalized by *N. glabrata* HGV20 than by *C. albicans* yeast cells.

A similar rationale can be applied to interpret *N. glabrata* HGV14 images (Figure 2, panels B1–B4). HGV14 strain also showed more intense labeling after incubation with the NE system. However, fewer HGV14 cells were labeled, even when applying the NE system, compared to HGV20. These results indicate a more consistent intracellular PS accumulation across the HGV20 cell population, even if some individual HGV14 cells displayed comparable labeling. Notably, incubation of the HGV11 strain with either ZnP-ethyl or NE system produced results comparable to those for HGV20 (Supporting Information, Figure S3). Interestingly, HGV20 and HGV11 presented a similar elliptical morphology with a size of approximately 6.5 × 4.0 μm, while HGV14 appeared rounder and smaller (ca. 4.5 × 4.0 μm).

### 3.3 Photodynamic Treatment of Planktonic Cells

PDI mediated by ZnP-ethyl or NE systems was investigated against planktonic cells of *N. glabrata* resistant strains. The results for the L+, AgNPs/L+, ZnP-ethyl/L–, and NE/L– groups are depicted in Figure 3A,3C, for the HGV20 and HGV14 strains, respectively. Yeasts exhibited minimal susceptibility to blue light alone (4.3 or 5.2 J/cm^2^). Furthermore, negligible susceptibility was observed in these cells after treatment with Zn­(II) porphyrin in the dark (ZnP-ethyl/L– group). Comparable responses were observed in *C. albicans* planktonic cells (ATCC 90028 and ATCC 10231) and *Leishmania* parasites, when exposed to similar light doses or Zn­(II) porphyrin concentrations (under dark conditions).^17–19^ Likewise, no effect was seen for the NE/L– group.

**Figure 3:**
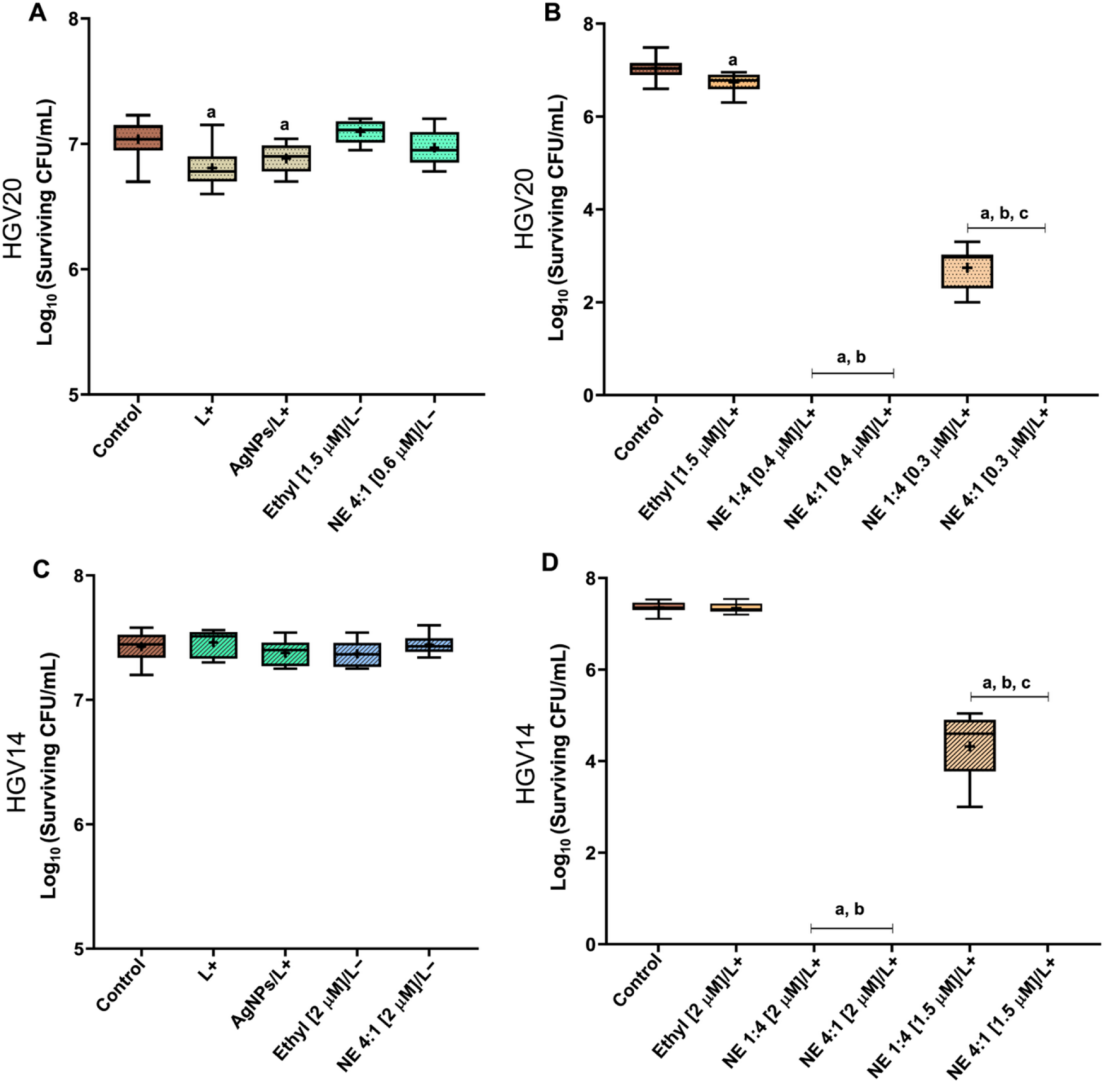
Box plots of resistant *N. glabrata* yeast cells HGV20 (A) and HGV14 (C) after incubation with ZnP-ethyl in the dark (L−) (at the highest concentration tested in NE), NE 4:1 (AgNPs/PS) in the dark (L−), or AgNPs/L+ (4:1, AgNPs/ultrapure water). Panels (A) and (C) also include yeast cells exposed to light alone (L+). Panels (B) and (D) present box plots of PDI-treated HGV20 and HGV14 yeasts, respectively. Ethyl/ZnP-ethyl, NE: AgNPs/ZnP-ethyl, Light dose: 4.3 J/cm^2^ for HGV20 and 5.2 J/cm^2^ for HGV14. The irradiation time was 3 min. The porphyrin concentration present in each system is shown in brackets. Control: yeasts neither irradiated nor treated, ^a^ *p* < 0.05 compared to the control, ^b^ *p* < 0.05 compared to ethyl groups, and ^c^ *p* < 0.05 between NE groups. The mean is indicated by a plus sign (+) in the box plots.

Additionally, AgNPs/L+ also induced a minimal effect on HGV20 yeast cells, likely due to the light action, as inferred from the NE/L– group results (which contains the same NP concentration). It is known that AgNPs can exert an antimicrobial effect, which can be influenced by their morphology, surface charge, interaction time, and microorganism type.^38^ Nevertheless, interaction time in the order of 10–13 min between AgNPs and *N. glabrata* cells was not sufficient to promote an effective CFU reduction, showing behavior comparable to that observed for *C. albicans* planktonic cells (ATCC 90028).^20^

The results of the photodynamic treatments (ZnP-ethyl/L+ and NE/L+ groups) are shown in Figure 3B,D, for the HGV20 and HGV14 strains, respectively. PDI against HGV20 yeast cells mediated by ZnP-ethyl led to a decrease of less than 1 log_10_ in CFU/mL compared to the control group, a result comparable to that of the L+ group. Conversely, the NE system with the higher proportion of AgNPs (4:1 v/v ratio) completely eradicated HGV20 cells, while the NE mixture at 1:4 (v/v ratio) resulted in an approximate 4 log_10_ reduction; both NE systems with 0.3 μM of PS (about 5× lower than the Zn­(II) porphyrin concentration applied alone). Moreover, complete eradication was observed for both proportions of the NE system when 0.4 μM PS was used.

Low susceptibility to PDI mediated by ZnP-ethyl alone was also observed against *C. albicans* planktonic cells (ATCC 90028 and ATCC 10231), even at higher PS concentration, using similar incubation and irradiation parameters.^18,20^ On the other hand, NE systems containing 0.6 μM porphyrin were able to eradicate *C. albicans* ATCC 90028 cells. Notably, that was twice the minimal lethal porphyrin concentration used in the NE 4:1 system for *N. glabrata* HGV20. Thus, HGV20 was more susceptible to NE-mediated PDI compared with the result previously reported for *C. albicans*, despite both species showing marked cellular accumulation of porphyrin facilitated by the NE system.

On the other hand, HGV14 cells were completely eradicated only following PDI mediated by NE 1:4 and 4:1 at 2 μM of porphyrin (or NE 4:1 at 1.5 μM), and at a high light dose of 5.2 J/cm^2^, a result that may be underpinned by the low cellular PS uptake by this strain. A higher light dose was applied to assess whether HGV14 yeast cells could be eradicated following NE incubation, as initial experiments indicated that eradication would not be achieved using the same parameters applied to the other strains. Interestingly, the HGV14 strain, which was defective in biofilm formation (Experimental Procedures), was more resistant to the applied PDI protocol. Ideally, PDI-induced ROS should be generated inside cells to promote more effective killing, which appears to have been boosted with NE, even though PS internalization had been lower in the HGV14 strain. However, although generally less effective, NE-enhanced ROS generated near the outer cell surface may also play an important role in the overall effects of PDI. Strains that are nonbiofilm producers, such as HGV14, may rely on alternative biological mechanisms to confer resistance, which may include cell wall remodeling—such as changes in charge, composition, and/or thickness—as well as metabolic and morphological adaptations.^39,40^ Cell wall alterations may involve modifications in polysaccharide composition, including mannans, chitin, and other glycoproteins.^41,42^ Such changes may influence the uptake of the compounds. Together, these factors may be related to the lower porphyrin internalization and decreased susceptibility to PDI observed in the HGV14 strain.

As with the cell interaction analysis, PDI of HGV11 yeasts yielded results comparable to those of HGV20. PDI with NE containing 0.4 μM porphyrin (4:1 and 1:4) reduced HGV11 CFU/mL in a similar proportion to that observed for HGV20 using NE at 0.3 μM PS, under the same irradiation parameters (Supporting Information, Figure S4).

Thus, systems containing ZnP-ethyl plus AgNPs, especially the one with higher proportion of nanostructures, were more effective in the PDI of resistant *N. glabrata* yeast cells than PS alone, likely due to an improved ROS production induced by the optical properties of AgNPs,^25^ as discussed in Section 3.1, and enhanced PS accumulation within cells, also promoted by these nanostructures (particularly in HGV20 and HGV11). The interaction of AgNPs with cellular structures is not governed by their optical properties, but rather by their physicochemical characteristics, including particle size and surface chemistry.^43^

### 3.4 Photodynamic Treatment of Biofilms

PDI mediated by the NE system (4:1) was also evaluated on mature biofilms of resistant *N. glabrata* applying MTT assay (Figure 4). This AgNPs/PS ratio was chosen due to its greater effect on planktonic cells. Regarding the HGV20 strain (Figure 4A), when biofilms were incubated with 1.5 μM of ZnP-ethyl in the dark, a negligible reduction in viability was observed. Biofilms of *C. albicans* (ATCC 90028) incubated with another *meso-N*-alkylpyridinium ZnP in the dark, Zn­(II) *meso*-tetrakis­(*N*-*n*-hexylpyridinium-2-yl)­porphyrin (ZnTnHex-2-PyP^4+^, ZnP-hexyl) also showed negligible viability reduction.^19^ On the other hand, biofilms incubated for 23 min with AgNPs or NE (4:1 v/v, 0.8 μM of PS), without irradiation (L−), caused a reduction of ca. 35% in viability. An effect of AgNPs on viability of *N. glabrata* (MCC-1152) biofilms was also observed by Ahamad and Fatma;^44^ however, in their study, biofilms were grown for 24 h and then incubated with the nanostructures for an additional 24 h.

**Figure 4:**
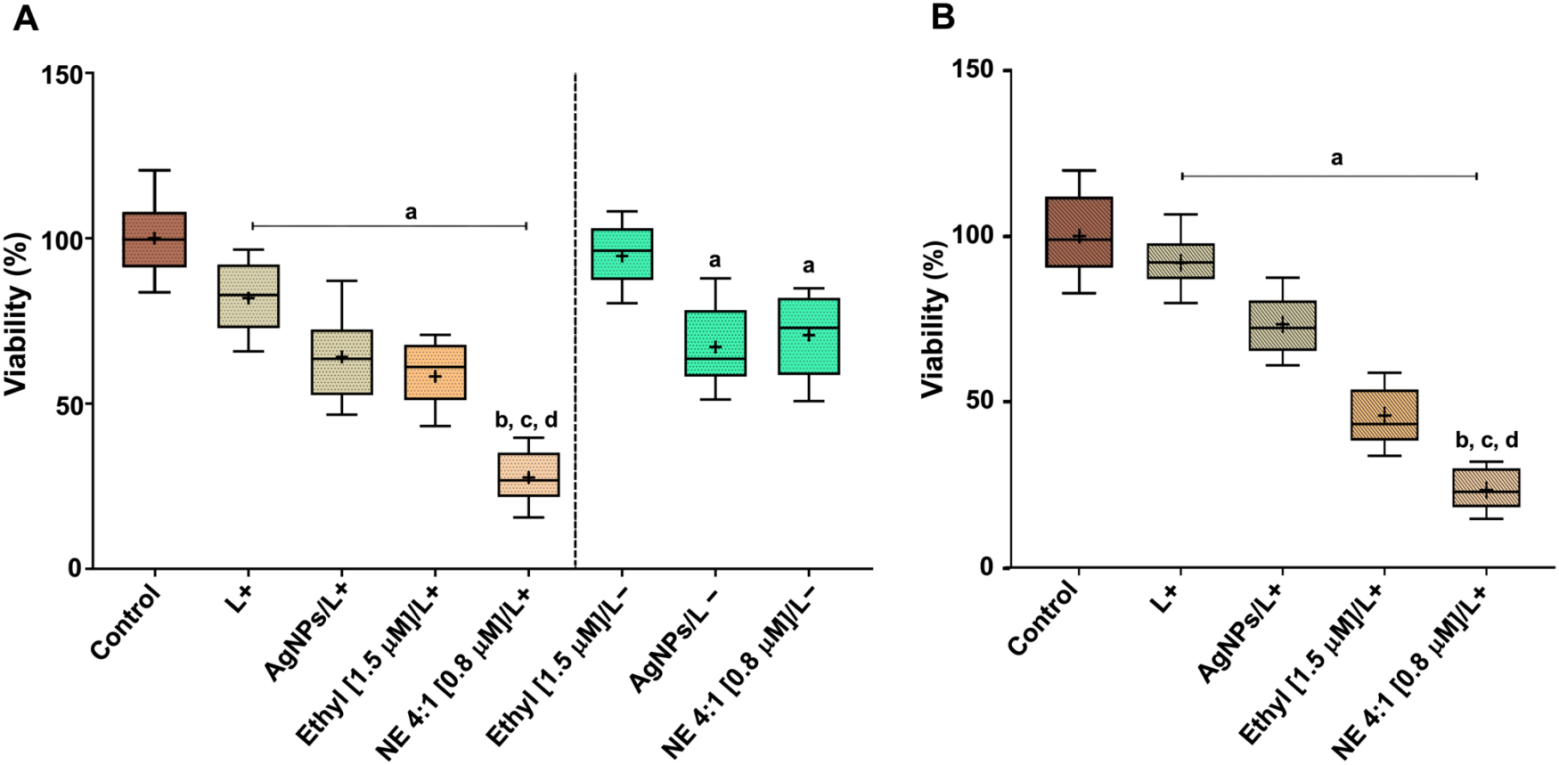
Box plots of viability of resistant *N. glabrata* HGV20 (A) and HGV11 (B) biofilms, assessed through MTT assay. Control: neither irradiated nor treated, L–: groups not irradiated, L+: groups irradiated (4.3 J/cm^2^). Ethyl: ZnP-ethyl, NE: AgNPs/ZnP-ethyl, and AgNPs (4:1, AgNPs/ultrapure water). The porphyrin concentration is shown in brackets. The irradiation time was 3 min, ^a^ *p* < 0.05 compared to the control, ^b,c,d^ *p* < 0.05 compared respectively to L+, AgNPs/L+, and ZnP-ethyl/L+. The mean is indicated by a plus sign (+) in the box plots.

Furthermore, biofilms irradiated only (4.3 J/cm^2^) presented a reduction in viability of about 15% compared with the control (untreated biofilm) for HGV20. A similar decrease was also observed in *C. albicans* (ATCC 90028) irradiated with the same light dose.^19^ This effect may be attributed to the presence of endogenous molecules in the biofilm that can act as PSs generating ROS.^45^ In addition, *N. glabrata* biofilms treated with 4:1/L+ AgNPs exhibited a similar reduction in viability to groups containing the nanostructures in the dark, which may be associated with the exposure time to AgNPs, as previously mentioned, suggesting that the effects of PS-like endogenous molecules and AgNPs alone were not additive under the analyzed conditions.

When HGV20 biofilms were photodynamically treated using ZnP-ethyl (1.5 μM), a reduction of approximately 40% in viability was observed. Davies and colleagues^46^ evaluated the cationic free-base porphyrin (i.e., a porphyrin without metal) TMP-1363 (5,10,15,20-tetrakis­(1-methylpyridinium-4-yl)­porphyrin tetra tosylate) as a PS in PDI of *N. glabrata* biofilms (ATCC MYA-275 and a clinical isolate). PDI (7.3 μM PS and light dose of 58.5 J/cm^2^) did not lead to a reduction in viability; a decrease was observed only when PDI was combined with miconazole treatment. Thus, we believe that our results may have the influence of the presence of Zn­(II), a diamagnetic metal, in the porphyrin structure. Zn can enhance the photophysical properties and cellular interaction of the Zn­(II) porphyrin, thereby contributing to improved ROS production and PDI performance,^47–49^ when compared with free-base porphyrins, as also observed with other Zn­(II) porphyrins, as ZnP-hexyl, in *C. albicans*.^19^ On the other hand, when biofilms were incubated with NE systems and irradiated, a greater reduction in viability was achieved, ca. 75% compared to the control, about double the decrease obtained in PDI mediated by ZnP-ethyl alone, while applying about half the PS concentration.

To complement and further understand the MTT assay results, biofilms treated with AgNPs alone/L+, ZnP-ethyl alone/L+, or NE/L+ were also analyzed by PI staining using confocal fluorescence microscopy. PI is a fluorescent dye that penetrates only cells with compromised membrane integrity, indicating cell death.^50^ As shown in Figure 5 (HGV20), cells from biofilms treated with NE/L+ were labeled to a much greater extent compared to other irradiated groups (including AgNPs alone/L+), even when about half of the porphyrin concentration was used. These results suggest that, although AgNPs/L+ may alter mitochondrial activity, it does not lead to substantial cell death. On the other hand, NE-mediated PDI not only resulted in the greatest reduction of viability but also in the highest cell death, as confirmed by PI staining. The data of L+ groups in HGV11 biofilms (Figures 4B and S5) followed a profile comparable to that of HGV20. Therefore, AgNPs associated with the ZnP-ethyl also enhanced PDI against biofilms, a notably more complex and resistant fungal structure.

**Figure 5:**
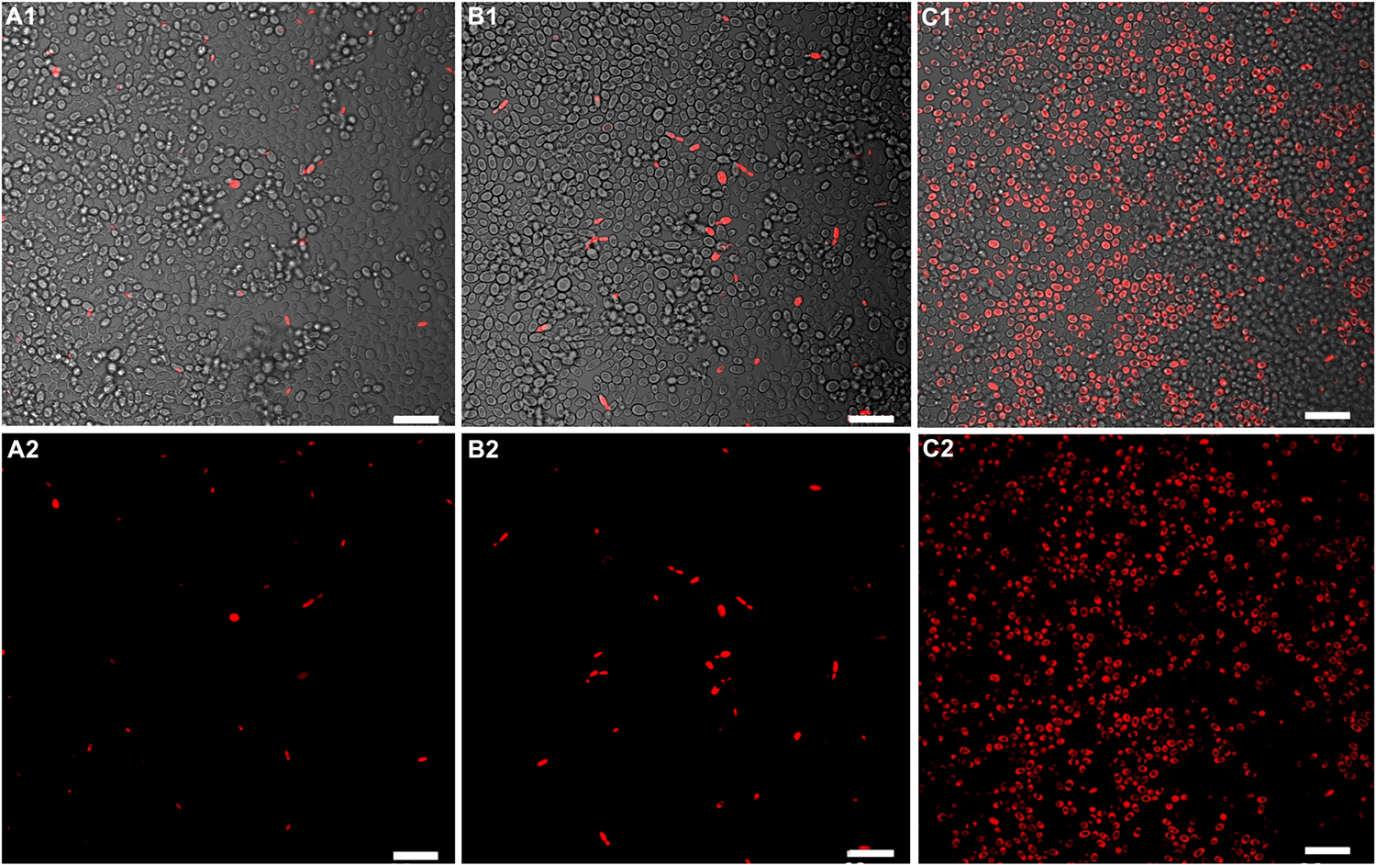
Representative confocal fluorescence microscopy images of HGV20 *N. glabrata* biofilms stained with PI after treatments: (A) AgNPs/L+ (4:1), (B) 1.5 μM ZnP-ethyl/L+, and (C) NE/L+ (4:1 and 0.8 μM porphyrin). Light dose: (4.3 J/cm^2^). Panels A1, B1, and C1 show overlays of bright-field and fluorescence channels. Scale bar = 20 μm.

In this light, we conclude that NE systems boosted PDI against resistant *N. glabrata* strains, either in the planktonic form or in biofilms. To the best of our knowledge, to date, no other studies have explored the plasmonic effect of metal NPs in PDI of *N. glabrata*.

## 4 Conclusion

The emergence of resistant isolates and the limited efficacy of antifungal agents have intensified the need to explore new alternatives for combating fungal infections, especially those caused by pathogens considered to be a high global priority. This study demonstrated that the combination of ZnP-ethyl porphyrin with PVP-AgNPs boosted the PDI of yeasts and biofilms from resistant strains of *N. glabrata*, using lower PS concentration and a short irradiation time—likely through the plasmonic effect and enhanced PS-cell interaction promoted by the nanostructures. Differences in PS uptake by yeasts and PDI susceptibility among strains may be interrelated. This study highlighted the potential of NE-mediated PDI against resistant *N. glabrata* strains and provided insights into the role of metal NPs in PDI, encouraging further research into metal NP-assisted PDI strategies to tackle mycoses and decontaminate medical devices.

## Supplementary Material



## Notes

Susceptibility assay methodology and antifungal susceptibility of *N. glabrata* strains (Table S1); size distribution of AgNPs obtained from TEM images (Figure S1); fluorescence lifetime curves of ZnP-ethyl and NE systems (Figure S2); confocal microscopy images of HGV11 yeast cells after incubation with ZnP-ethyl or NE system (Figure S3); PDI of HGV11 planktonic cells (Figure S4); and confocal microscopy images of HGV11 biofilms labeled with propidium iodide after photodynamic treatment (Figure S5) (PDF)
