# Supplementary material for: Improved Photodynamic Inactivation of Resistant Nakaseomyces glabrata Yeasts and Biofilms Mediated by ZnTE-2-PyP4+ Porphyrin Combined with Silver Nanoparticles
Source: ACS Omega. 2026 Feb 18;11(8):13435–44. doi: 10.1021/acsomega.5c10859 (PMC12961472; doi:10.1021/acsomega.5c10859)
Supplement: Supplementary file 1 [file ao5c10859_si_001.pdf]

Supporting Information

Improved Photodynamic Inactivation of Resistant *Nakaseomyces glabrata* Yeasts and Biofilms Mediated by ZnTE-2-PyP^{4+} Porphyrin Combined with Silver Nanoparticles

Geyse S. de Lima[a], Sueden O. Souza[a], Jacqueline C. Bueno-Janice[a], Bruno L. Raposo[a,b], Franz A. G. dos Santos[c], Rejane P. Neves[c], Beate S. Santos[d], Jose F. Sarmento-Neto[e], Julio S. Reboucas[e], Paulo E. Cabral Filho[a,*], Adriana Fontes[a,*]

[a] Departamento de Biofísica e Radiobiologia, Universidade Federal de Pernambuco, Recife, 50670-901, PE, Brazil.

[b] Centro de Lasers e Aplicações, Instituto de Pesquisas Energéticas e Nucleares (IPEN-CNEN/SP), São Paulo, 05508-000, SP, Brazil.

[c] Departamento de Micologia, Universidade Federal de Pernambuco, Recife, 50760-420, PE, Brazil.

[d] Departamento de Ciências Farmacêuticas, Universidade Federal de Pernambuco, Recife, 50740-520, PE, Brazil.

[e] Departamento de Química, Universidade Federal da Paraíba, João Pessoa, 58051-900, PB, Brazil.

***Corresponding authors:** Adriana Fontes and Paulo E. Cabral Filho, Departamento de Biofísica e Radiobiologia, Universidade Federal de Pernambuco, Recife, 50670-901, PE, Brazil, Email: adriana.fontes@ufpe.br and Email: paulo.euzebio@ufpe.br.

1 – Susceptibility Assay

The broth microdilution method was employed to evaluate the antifungal susceptibility of the isolates, in accordance with the guidelines established in M27-A3 and M27-S4 by the Clinical and Laboratory Standards Institute (CLSI) [1,2]. For susceptibility test, the following compounds were prepared in dimethyl sulfoxide (DMSO, ACS Científica) at a stock concentration of 1600 g/mL: caspofungin (MSD), anidulafungin (Pfizer), micafungin (Astellas Pharma Inc.), itraconazole (Sigma-Aldrich), voriconazole (Sigma-Aldrich), fluconazole (Pfizer), ketoconazole (Sigma-Aldrich), and amphotericin B (Sigma-Aldrich). These compounds were then diluted in the isolates at concentrations ranging from 0.03 to 16 µg/mL. The subculture of *N. glabrata* isolates in SDA was performed in a manner consistent with established protocols, with a minimum of three colonies being collected for analysis. The optical density (OD) at 530 nm was then adjusted in a saline suspension (0.85 g/L NaCl) to achieve a range of 1-5×10^6 CFU/mL, thereby ensuring 90% transmittance. The inoculum was added to 96-well plates using RPMI 1640 medium containing 0.165 M 3-Morpholinopropane sulfonic acid (MOPS) (Sigma-Aldrich). The samples were then incubated with the respective compounds for 24 h at 37 °C to determine the minimum inhibitory concentration (MIC) based on visual inspection and OD_{530}. These tests were carried out in duplicate for all isolates, and the results are presented in Table S1.

(1) Clinical and Laboratory Standards Institute. Reference method for broth dilution antifungal susceptibility testing of yeasts; approved standard, 3rd ed. CLSI document M27-A3. CLSI, Wayne, PA, 2008.

(2) Clinical and Laboratory Standards Institute. Reference method for broth dilution antifungal susceptibility testing of yeasts; CLSI document M27-S4. CLSI, Wayne, PA, 2012.

Table S1. Antifungal susceptibility of *N. glabrata* strains.

N. glabrata	**Susceptibility (µg/mL)**							
	FLU	ITRA	VORI	KETO	MICA	ANID	CASPO	AMPB
**HGV11**	1	16	16	2	0.03	0.06	0.06	0.5
**HGV14**	1	0.03	0.25	0.03	0.03	0.06	0.5	0.25
**HGV20**	1	0.125	0.25	0.03	0.03	0.5	0.5	1

Legend (cell colours): green = Sensitive; yellow = Dose-dependent; orange = Resistant

FLU = Fluconazole, ITRA = Itraconazole, VORI = Voriconazole, KETO = Ketoconazole, MICA = Micafungin, ANID = Anidulafungin, CASPO = Caspofungin, AMPB = Amphotericin B.

2 – Characterizations of AgNPs and NE Systems

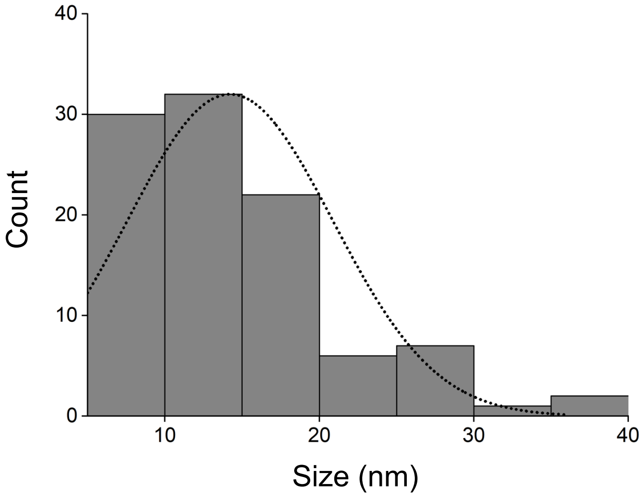

Figure S1. Particle size distribution obtained from transmission electron microscopy (TEM) images (total particles measured = 100).

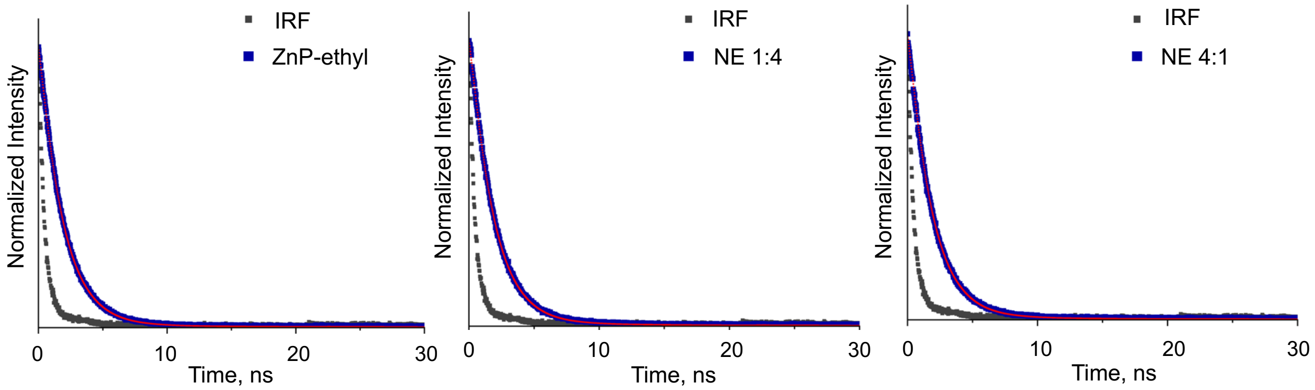

Figure S2. Fluorescence lifetime curves. IRF: Instrument response function, NE: AgNPs/ZnP-ethyl.

3 – Cell Interaction Analyses (HGV11)

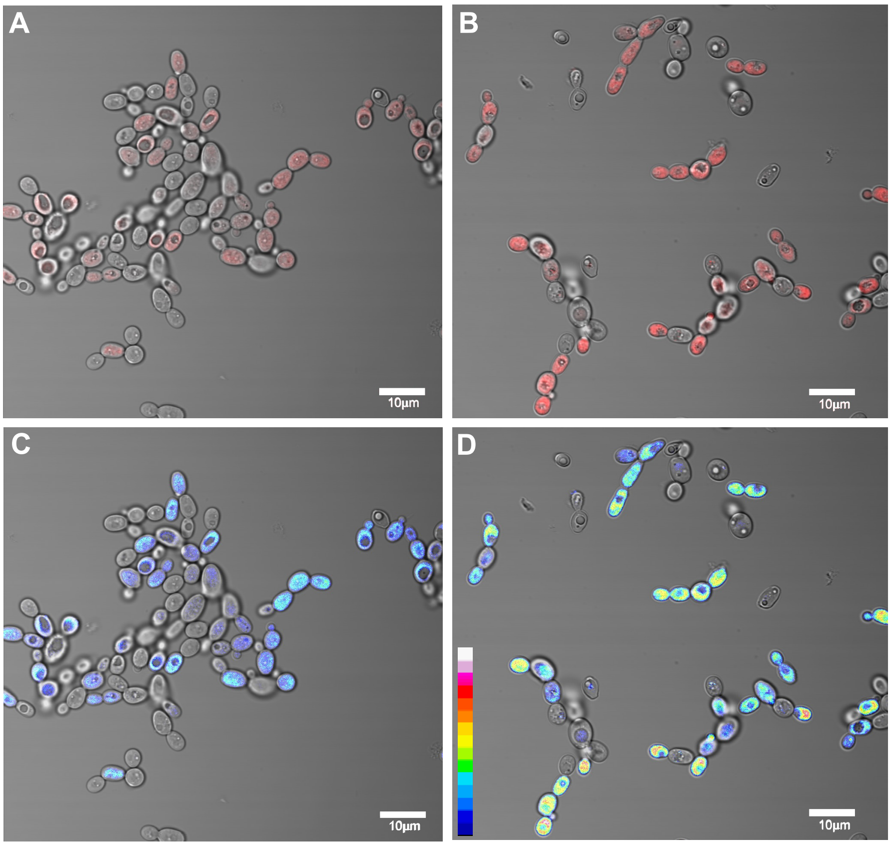

Figure S3. Representative confocal microscopy images of *N. glabrata* (HGV11) yeast cells, showing the overlay of bright-field and fluorescence channels after 10 min of incubation with (A) ZnP-ethyl or (B) NE 1:4 system. In (C) and (D) are the corresponding heatmaps, where the signal intensity increases from dark blue to white. Scale Bar = 10 µm.

4 – Photodynamic Inactivation (PDI) of HGV11 Planktonic Cells

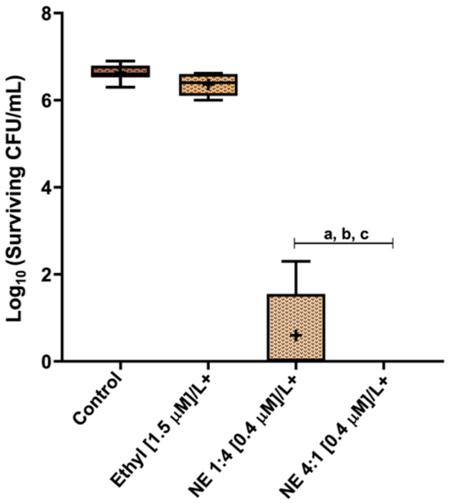

Figure S4. Box plots of PDI against *N. glabrata* HGV11 yeast cells. Ethyl: ZnP-ethyl, NE: AgNPs/ZnP-ethyl, Light dose: 4.3 J/cm^2. The porphyrin concentration is shown in brackets The irradiation time was 3 min. Control: yeast cells neither irradiated nor treated, $^a p < 0.05$ compared to the control, $^b p < 0.05$ compared to ethyl group, and $^c p < 0.05$ between NE groups. The mean is indicated by a plus sign (+) in the box plots.

5 – Propidium Iodide (PI) Labeling after Photodynamic Treatment (HGV11)

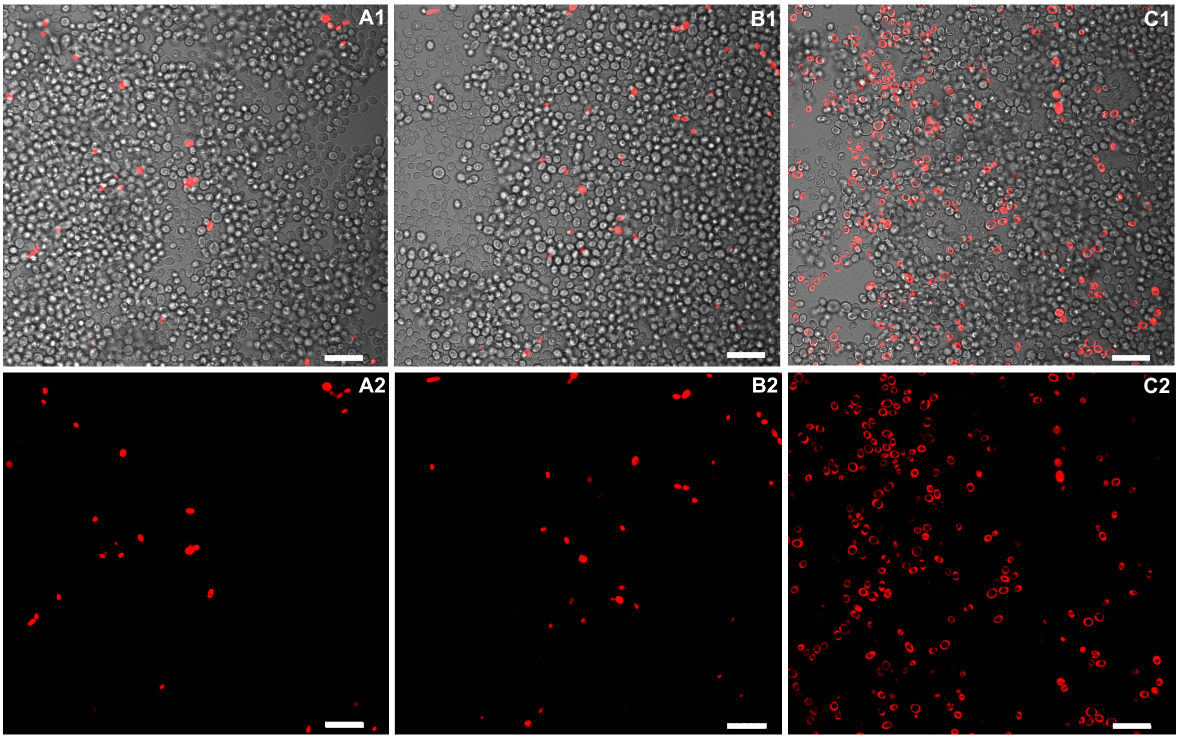

Figure S5. Representative confocal microscopy images of HGV11 *N. glabrata* biofilms stained with PI after treatments. (A) AgNPs/L+ (4:1), (B) ZnP-ethyl/L+, 1.5 µM of PS, and (C) NE/L+ (4:1 and 0.8 µM of porphyrin). Light dose: (4.3 J/cm^2). Panels A1, B1, and C1 show overlays of bright-field and fluorescence channels. Scale bar = 20 µm.
